# Impact of vancomycin therapeutic drug monitoring on mortality in sepsis patients across different age groups: a propensity score-matched retrospective cohort study

**DOI:** 10.3389/fmed.2024.1498337

**Published:** 2024-12-12

**Authors:** Huaidong Peng, Ruichang Zhang, Shuangwu Zhou, Tingting Xu, Ruolun Wang, Qilin Yang, Xunlong Zhong, Xiaorui Liu

**Affiliations:** ^1^Department of Pharmacy, The Second Affiliated Hospital, Guangzhou Medical University, Guangzhou, China; ^2^Department of Critical Care, Guangzhou Twelfth People' Hospital, Guangzhou, China; ^3^The Second School of Clinical Medicine, Guangzhou Medical University, Guangzhou, China; ^4^School of Pharmaceutical Sciences, Guangzhou Medical University, Guangzhou, China; ^5^Department of Critical Care, The Second Affiliated Hospital, Guangzhou Medical University, Guangzhou, China; ^6^Department of Pharmacy, Guangzhou Institute of Cancer Research, The Affiliated Cancer Hospital, Guangzhou Medical University, Guangzhou, China

**Keywords:** vancomycin, therapeutic drug monitoring, sepsis, mortality, age

## Abstract

**Background:**

Due to its potent antibacterial activity, vancomycin is widely used in the treatment of sepsis. Therapeutic drug monitoring (TDM) can optimize personalized vancomycin dosing regimens, enhancing therapeutic efficacy and minimizing nephrotoxic risk, thereby potentially improving patient outcomes. However, it remains uncertain whether TDM affects the mortality rate among sepsis patients or whether age plays a role in this outcome.

**Methods:**

We analyzed data from the Medical Information Mart of Intensive Care–IV database, focusing on sepsis patients who were admitted to the intensive care unit (ICU) and treated with vancomycin. The primary variable of interest was the use of vancomycin TDM during the ICU stay. The primary outcome was 30-day mortality. To control for potential confounding factors and evaluate associations, we used Cox proportional hazards regression and propensity score matching (PSM). Subgroup and sensitivity analyses were performed to assess the robustness of our findings. Furthermore, restricted cubic spline models were utilized to investigate the relationship between age and mortality among different groups of sepsis patients, to identify potential non-linear associations.

**Results:**

A total of 14,053 sepsis patients met the study criteria, of whom 6,826 received at least one TDM during their ICU stay. After PSM, analysis of 4,329 matched pairs revealed a significantly lower 30-day mortality in the TDM group compared with the non-TDM group (23.3% vs.27.7%, *p* < 0.001). Multivariable Cox proportional hazards regression showed a significantly reduced 30-day mortality risk in the TDM group [adjusted hazard ratio (HR): 0.66; 95% confidence interval (CI): 0.61–0.71; *p* < 0.001]. This finding was supported by PSM-adjusted analysis (adjusted HR: 0.71; 95% CI: 0.66–0.77; *p* < 0.001) and inverse probability of treatment weighting analysis (adjusted HR: 0.72; 95% CI: 0.67–0.77; *p* < 0.001). Kaplan–Meier survival curves also indicated significantly higher 30-day survival in the TDM group (log-rank test, *p* < 0.0001). Subgroup analyses by gender, age, and race yielded consistent results. Patients with higher severity of illness—indicated by sequential organ failure assessment scores ≥6, acute physiology score III ≥40, or requiring renal replacement therapy, vasopressors, or mechanical ventilation—experienced more pronounced mortality improvement from vancomycin TDM compared with those with lower severity scores or not requiring these interventions. The results remained robust after excluding patients with ICU stays <48 h, those with methicillin-resistant *Staphylococcus aureus* infections, or when considering only patients with septic shock. In subgroup analyses, patients under 65 years (adjusted HR: 0.50; 95% CI: 0.43–0.58) benefited more from vancomycin TDM than those aged 65 years and older (adjusted HR: 0.75; 95% CI: 0.67–0.83). Notably, sepsis patients aged 18–50 years had the lowest mortality rate among all age groups, at 15.2% both before and after PSM. Furthermore, in this age group, vancomycin TDM was associated with a greater reduction in 30-day mortality risk, with adjusted HRs of 0.32 (95% CI: 0.24–0.41) before PSM and 0.30 (95% CI: 0.22–0.32) after PSM.

**Conclusion:**

Vancomycin TDM is associated with reduced 30-day mortality in sepsis patients, with the most significant benefit observed in patients aged 18–50. This age group exhibited the lowest mortality rates and experienced the greatest reduction in mortality following TDM compared with older patients.

## 1 Introduction

Sepsis is a life-threatening condition resulting from a dysregulated host response to infection, accounting for ~30% of global intensive care unit (ICU) admissions (1, 2). It is estimated that there are 49 million cases of sepsis worldwide each year, resulting in 11 million deaths (3). Currently, sepsis is a leading cause of rising healthcare costs and in-hospital mortality rates (3, 4). Therefore, timely antimicrobial treatment is crucial for patients suspected of or diagnosed with sepsis (5). Vancomycin is a glycopeptide antibiotic with potent bactericidal activity against Gram-positive cocci. It is the primary drug used to treat methicillin-resistant *Staphylococcus aureus* (MRSA) infections (6, 7). According to the Surviving Sepsis Guidelines, MRSA coverage is recommended in the initial management of high-risk sepsis and septic shock patients (8). Empirical use of vancomycin to address potential MRSA or other drug-resistant Gram-positive bacterial infections is a common and necessary practice in the initial treatment of sepsis (5). The vancomycin dosing regimen should be guided by its pharmacokinetic (PK) and pharmacodynamic (PD) properties, with adjustments based on therapeutic drug monitoring (TDM) to manage its narrow therapeutic index and address growing resistance challenges (9, 10). Mounting evidence has demonstrated significant differences in PK/PD among sepsis patients, making TDM crucial for maximizing vancomycin efficacy while minimizing adverse effects (11–13). It is generally recommended to monitor trough concentrations (targeting 10–20 μg/ml) or the area under the curve (AUC)/minimum inhibitory concentration (MIC) ratio (targeting 400–600) (9, 10, 14). In summary, TDM and personalized dose adjustments have been shown to improve therapeutic outcomes and are expected to enhance patient prognosis (15).

In vancomycin TDM, assessing the AUC generally requires 1–2 blood concentration measurements (14, 16, 17). Thus, by verifying if blood concentration measurements were performed, we can identify patients who underwent vancomycin TDM, regardless of whether the monitored parameter was directly measured trough concentration or the calculated AUC. However, only a few retrospective studies have explored the impact of implementing TDM on clinical outcomes (18–21). Research has shown that vancomycin TDM can improve therapeutic efficacy and reduce nephrotoxicity (22), but its impact on mortality, particularly in sepsis patients, remains inadequately studied (23). In clinical practice, vancomycin is often used as empiric treatment for sepsis patients, and the benefits of TDM remain uncertain when the pathogen is not identified. Moreover, TDM requires significant medical resources and adds to treatment costs (24), necessitating a clear demonstration of its benefits. Sepsis is a highly heterogeneous disease, and mortality rates are closely related to patient age (25–27). However, differences in mortality rates among sepsis patients receiving vancomycin TDM across different age groups remain unclear. Therefore, we designed this study to investigate the impact of vancomycin TDM on mortality in sepsis patients and to analyze whether this effect varies across different age groups.

## 2 Materials and methods

### 2.1 Data source and ethics approval

The data for this study were sourced from the Medical Information Mart for Intensive Care (MIMIC)-IV database (version 2.2). MIMIC-IV is an open-access, real-world clinical database that includes hospitalization data for over 70,000 ICU patients at Beth Israel Deaconess Medical Center from 2008 to 2019 (28). The use of this database was approved by the Institutional Review Board of Beth Israel Deaconess Medical Center (Project Number: 2001-P-001699/14), with informed consent waived. Our research team completed the required training and was granted access credentials (Certificate Number: 59679596) for using the database. This study complies with the ethical principles and reporting standards specified in the Strengthening the Reporting of Observational Studies in Epidemiology (STROBE) guidelines (29).

### 2.2 Patient selection criteria

**Figure 1:**
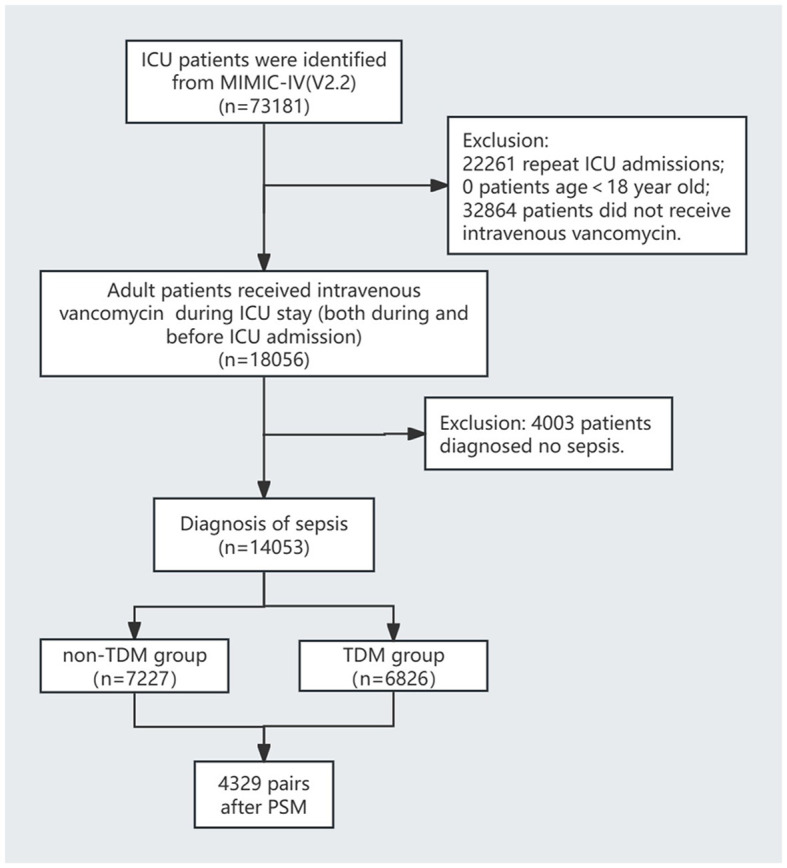
Flowchart of included patients.

The inclusion criteria were: (1) admission to the ICU; (2) administration of intravenous vancomycin during the ICU stay, regardless of when the treatment started; and (3) diagnosis of sepsis according to the Sepsis-3 criteria, defined by a documented or suspected infection and a sequential organ failure assessment (SOFA) score of ≥2 (1). The exclusion criteria were: (1) patients who were not on their first ICU admission; (2) no intravenous vancomycin treatment during the ICU stay; (3) age < 18 years; and (4) not meeting the sepsis diagnostic criteria. The patient selection process is illustrated in Figure 1.

### 2.3 Exposure and outcomes

The primary exposure variable in this study was whether vancomycin TDM was conducted during the ICU stay. Vancomycin TDM was defined as the measurement of at least one serum concentration —whether trough, peak, or random level—during the ICU stay. Patients who underwent vancomycin TDM were classified into the TDM group, while those who did not were categorized into the non-TDM group.

The primary outcome of this study was 30-day mortality. Secondary outcomes included ICU mortality, in-hospital mortality, length of ICU stay, and total hospital stay.

### 2.4 Data extraction

Patient data were extracted from the MIMIC-IV database using Structured Query Language (SQL). The corresponding SQL script used for data extraction is available on GitHub (https://github.com/MIT-LCP/mimic-iv). The extracted data included variables such as baseline demographics, vital signs, general laboratory tests, comorbidities, disease severity scores, treatment interventions, and vancomycin TDM details. A comprehensive list of extracted variables can be found in Table 1.

**Table 1:** Baseline characteristics of the patients enrolled from the MIMIC-IV database.

**Patient characteristic**	**Before PSM**				**After PSM**			
	**Total (*****n*** = **14,053)**	**Non-TDM group (*****n*** = **7,227)**	**TDM group (*****n*** = **6,826)**	**SMD**	**Total (*****n*** = **8,658)**	**Non-TDM group (*****n*** = **4,329)**	**TDM group (*****n*** = **4,329)**	**SMD**
Gender [male, *n* (%)]	8,197 (58.3)	4,153 (57.5)	4,044 (59.2)	0.036	3,654 (42.2)	1,835 (42.4)	1,819 (42)	0.007
Age (years)	66.2 ± 16.3	67.8 ± 15.9	64.6 ± 16.5	0.200	66.2 ± 16.5	66.3 ± 16.6	66.2 ± 16.4	0.005
RACE [white, *n* (%)]	9,168 (65.2)	4,907 (67.9)	4,261 (62.4)	0.115	5,579 (64.4)	2,794 (64.5)	2,785 (64.3)	0.004
**Vital signs**								
Heart rate (bpm)	88.2 ± 16.5	86.6 ± 15.6	89.8 ± 17.2	0.196	88.4 ± 16.6	88.4 ± 16.4	88.3 ± 16.7	0.004
MAP (mmHg)	76.1 ± 10.2	75.7 ± 10.0	76.5 ± 10.4	0.076	76.3 ± 10.4	76.3 ± 10.6	76.3 ± 10.3	0.003
Respiratory rate (/min)	20.2 ± 4.2	19.7 ± 4.0	20.8 ± 4.3	0.271	20.2 ± 4.2	20.2 ± 4.2	20.2 ± 4.1	0.015
Temperature (°C)	37.6 ± 0.9	37.5 ± 0.8	37.7 ± 0.9	0.240	37.6 ± 0.9	37.6 ± 0.9	37.6 ± 0.9	0.014
SpO_2_ (%)	96.8 ± 2.6	96.8 ± 2.6	96.8 ± 2.5	0.002	96.8 ± 2.6	96.8 ± 2.7	96.8 ± 2.4	0.004
**Laboratory tests**								
WBC (× 10^9^)	14.5 (10.3, 19.8)	14.2 (10.1, 19.0)	14.9 (10.5, 20.5)	0.079	14.6 (10.3, 19.9)	14.6 (10.4, 19.7)	14.6 (10.3, 20.1)	0.001
Hemoglobin (g/L)	9.9 ± 2.2	9.8 ± 2.1	9.9 ± 2.2	0.013	9.9 ± 2.2	9.9 ± 2.2	9.9 ± 2.2	0.003
Hematocrit (%)	29.8 ± 6.5	29.6 ± 6.3	30.0 ± 6.7	0.053	29.9 ± 6.6	29.9 ± 6.6	29.9 ± 6.6	0.002
Platelets (× 10^9^)	161.0 (108.0, 227.0)	158.0 (110.0, 220.0)	165.0 (105.2, 233.0)	0.061	164.0 (109.0, 232.0)	164.0 (112.0, 230.0)	164.0 (107.0, 233.0)	0.002
Creatinine (mg/dL)	1.2 (0.9, 2.0)	1.1 (0.8, 1.6)	1.3 (0.9, 2.4)	0.320	1.2 (0.9, 2.0)	1.2 (0.9, 1.9)	1.2 (0.8, 2.0)	0.023
BUN (mg/dL)	24.0 (16.0, 40.0)	22.0 (15.0, 34.0)	27.0 (17.0, 46.0)	0.298	24.0 (16.0, 41.0)	24.0 (16.0, 40.0)	25.0 (16.0, 41.0)	0.012
Glucose (finger, mg/dL)	133.5 (114.8, 164.3)	131.5 (115.0, 157.1)	136.5 (114.5, 171.8)	0.051	134.7 (114.7, 167.3)	134.9 (116.0, 167.4)	134.4 (114.0, 167.2)	0.003
Potassium (mmol/L)	3.9 ± 0.6	3.9 ± 0.6	3.9 ± 0.6	0.051	3.9 ± 0.6	3.9 ± 0.6	3.9 ± 0.6	0.005
Bicarbonate (mmol/L)	20.6 ± 5.1	21.0 ± 4.9	20.1 ± 5.4	0.172	20.6 ± 5.2	20.5 ± 5.4	20.6 ± 5.1	0.006
**Comorbidity diseases**, ***n*** **(%)**								
Hypertension	8,776 (62.4)	4,618 (63.9)	4,158 (60.9)	0.062	5,388 (62.2)	2,690 (62.1)	2,698 (62.3)	0.004
Congestive heart failure	4,249 (30.2)	2,109 (29.2)	2,140 (31.4)	0.047	2,707 (31.3)	1,365 (31.5)	1,342 (31)	0.011
COPD	3,729 (26.5)	1,847 (25.6)	1,882 (27.6)	0.046	2,334 (27.0)	1,188 (27.4)	1,146 (26.5)	0.022
Liver disease	2,307 (16.4)	945 (13.1)	1,362 (20)	0.186	1,400 (16.2)	704 (16.3)	696 (16.1)	0.005
Diabetes	3,463 (24.6)	1,794 (24.8)	1,669 (24.5)	0.009	2,117 (24.5)	1,058 (24.4)	1,059 (24.5)	0.001
Renal disease	3,103 (22.1)	1,424 (19.7)	1,679 (24.6)	0.118	2,004 (23.1)	992 (22.9)	1,012 (23.4)	0.011
Malignant cancer	2,048 (14.6)	1,063 (14.7)	985 (14.4)	0.008	1,298 (15.0)	649 (15)	649 (15)	< 0.001
Cerebrovascular disease	2,039 (14.5)	898 (12.4)	1,141 (16.7)	0.122	1,325 (15.3)	664 (15.3)	661 (15.3)	0.002
**Severity of illness scores**								
CCI	5.9 ± 2.9	5.9 ± 2.9	6.0 ± 3.0	0.035	6.0 ± 3.0	6.0 ± 3.0	6.0 ± 3.0	0.008
SOFA score	6.0 (4.0, 8.0)	5.0 (3.0, 7.0)	7.0 (4.0, 9.0)	0.398	6.0 (4.0, 8.0)	6.0 (4.0, 8.0)	6.0 (4.0, 8.0)	0.016
APS III	60.4 ± 27.2	52.4 ± 24.5	68.8 ± 27.3	0.634	60.0 ± 24.8	60.0 ± 26.0	60.0 ± 23.5	0.001
SAPS II	41.6 ± 15.1	39.7 ± 14.7	43.7 ± 15.1	0.267	41.9 ± 15.1	42.1 ± 15.9	41.8 ± 14.2	0.021
OASIS	36.4 ± 9.5	33.9 ± 9.0	39.0 ± 9.3	0.562	36.6 ± 8.9	36.7 ± 9.0	36.5 ± 8.8	0.015
**Therapy**, ***n*** **(%)**								
RRT	856 (6.1)	231 (3.2)	625 (9.2)	0.249	453 (5.2)	213 (4.9)	240 (5.5)	0.028
Mechanical ventilation	8,620 (61.3)	3,595 (49.7)	5,025 (73.6)	0.507	5,444 (62.9)	2,733 (63.1)	2,711 (62.6)	0.011
Vasoactive drug	8,236 (58.6)	3,786 (52.4)	4,450 (65.2)	0.262	4,939 (57.0)	2,486 (57.4)	2,453 (56.7)	0.015
**Infectious pathogen**, ***n*** **(%)**								
MRSA	1,135 (8.1)	423 (5.9)	712 (10.4)	0.168	708 (8.2)	351 (8.1)	357 (8.2)	0.005

MAP, mean arterial pressure; SpO_2_, percutaneous arterial oxygen saturation; WBC, white blood cell count; BUN, blood urea nitrogen; COPD, chronic obstructive pulmonary disease; CCI, charlson comorbidity score; SOFA score, sequential organ failure score; APS III, acute physiology score III; SAPS II, simplified acute physiology score II; OASIS, oxford acute severity of illness score; RRT, renal replacement therapy; MRSA, methicillin-resistant Staphylococcus aureus.

### 2.5 Statistical analysis

The sample size for this study was determined by the available dataset. The extracted data were initially preprocessed, and missing values were handled using the K-Nearest Neighbors imputation method (30). Detailed information on missing data is available in Supplementary Table 1. Continuous variables following a normal or approximately normal distribution were presented as mean ± standard deviation (SD), while non-normally distributed variables were presented as median and interquartile range. Categorical variables were expressed as frequencies (percentages). Between-group comparisons of continuous variables were conducted using the Student's *t*-test or the Wilcoxon rank-sum test. Categorical variables were compared using Pearson's chi-square test or Fisher's exact test, depending on specific conditions.

To minimize selection bias from potential confounders, propensity score matching was employed using a logistic regression model with 1:1 nearest-neighbor matching and a caliper of 0.1. Variables included in the PSM model were selected based on previous literature and included age, sex, race, laboratory results, comorbidities, severity scores, and treatment interventions. Covariate balance between groups was evaluated using the standardized mean difference (SMD), with an SMD of < 10% after PSM indicating well-balanced groups.

To assess the association between vancomycin TDM and 30-day mortality, Cox proportional hazards regression analysis was performed, adjusting for confounders listed in Table 1. Survival analysis was conducted using the Kaplan–Meier method, and between-group differences were evaluated by the log-rank test. Following PSM, subgroup analyses were performed to evaluate the association between vancomycin TDM and 30-day mortality across various subgroups. Sensitivity analyses were performed by excluding patients with an ICU stay of < 48 h or those diagnosed with MRSA infection. Additionally, further validation was performed among patients with septic shock.

Specifically, the relationship between age and 30-day mortality was investigated. The restricted cubic spline (RCS) model was applied to explore potential nonlinear associations between age and 30-day mortality. Age was further stratified to analyze the impact of vancomycin TDM on 30-day mortality in sepsis patients across different age groups.

All statistical analyses were performed using R statistical software (version 3.3.2, https://www.R-project.org) and Free Statistics software (version 1.9.1, https://www.clinicalscientists.cn/freestatistics/). A two-sided test was employed, with *p* < 0.05 considered statistically significant.

## 3 Results

### 3.1 Demographic and clinical information in patients before PSM

The flow diagram of this study is shown in Figure 1. This study included 14,053 sepsis patients from the MIMIC-IV database who were admitted to the ICU and received intravenous vancomycin. Among them, 6,826 patients (48.6%) underwent at least one vancomycin serum concentration measurement, while the remaining 7,227 patients (51.4%) did not (Table 1). Before PSM, significant differences were observed across most variables (*p* < 0.05), except for peripheral oxygen saturation (SpO_2_), hemoglobin levels, and the proportions of patients with diabetes and malignant tumors. Although patients in the TDM group were younger than those in the non-TDM group (*p* < 0.001), their scores on all five severity indices were significantly higher (*p* < 0.001). Additionally, the TDM group had significantly higher proportions of patients with comorbidities such as congestive heart failure, chronic obstructive pulmonary disease (COPD), liver disease, renal disease, and cerebrovascular disease (*p* < 0.001). Compared with the non-TDM group, the TDM group also had significantly higher need for renal replacement therapy (RRT), vasopressor support, and mechanical ventilation (*p* < 0.001). These data indicate that patients in the TDM group presented with more complex and severe conditions than those in the non-TDM group.

In terms of clinical outcomes, the 30-day mortality rate (27.2% vs. 16.2%; *p* < 0.001), ICU mortality (17.8% vs. 12.8%; *p* < 0.001), and in-hospital mortality (23.7% vs. 16.8%; *p* < 0.001) were significantly higher in the TDM group. Furthermore, patients in the TDM group had significantly longer ICU stays compared with those in the non-TDM group [median 6.9 days, interquartile range (IQR) 3.7–12.8 vs. median 2.2 days, IQR 1.3–3.9; *p* < 0.001]. Similarly, total hospital stays were also longer in the TDM group (median 14.1 days, IQR 8.5–22.9) compared with the non-TDM group (median 7.5 days, IQR 4.9–11.8; *p* < 0.001; Table 2).

**Table 2:** Primary outcome and secondary outcomes of the study.

**Outcomes**	**Matching**	**Total**	**Non-TDM group**	**TDM group**	***p***
30 days mortality	Before PSM, *n* (%)	3,350/14,053 (23.8)	1,494/7,227 (16.2)	1,856/6,826 (27.2)	< 0.001
	After PSM, *n* (%)	2,211/8,658 (25.5)	1,201/4,329 (27.7)	1,010/4,329 (23.3)	< 0.001
ICU mortality	Before PSM, *n* (%)	2,138/14,053 (15.2)	924/7,227 (12.8)	1,214/6,826 (17.8)	< 0.001
	After PSM, *n* (%)	1,375/8,658 (15.9)	810/4,329 (18.7)	565/4,329 (13.1)	< 0.001
Hospital mortality	Before PSM, *n* (%)	2,830/14,053 (20.1)	1,214/7,227 (16.8)	1,616/6,826 (23.7)	< 0.001
	After PSM, *n* (%)	1,824/8,658 (21.1)	1,012/4,329 (23.4)	812/4,329 (18.8)	< 0.001
The length of ICU stay (days)	Before PSM, *n* (%)	3.7 (1.9, 8.0)	2.2 (1.3, 3.9)	6.9 (3.7, 12.8)	< 0.001
	After PSM, *n* (%)	3.8 (2.0, 7.4)	2.6 (1.5, 4.7)	5.6 (3.1, 10.6)	< 0.001
The length of hospital stay (days)	Before PSM, *n* (%)	9.9 (5.9, 17.4)	7.5 (4.9, 11.8)	14.1 (8.5, 22.9)	< 0.001
	After PSM, *n* (%)	10.0 (5.9, 17.1)	7.9 (4.7, 12.8)	13.0 (8.0, 21.2)	< 0.001

### 3.2 Demographic and clinical information in patients after PSM

In the PSM analysis, 4,329 patient pairs were successfully matched between the TDM and non-TDM groups. After PSM, there were no notable differences in baseline characteristics between the two groups (Table 1). Table 2 presents the clinical outcomes following PSM. The overall 30-day mortality rate was 25.5% (2,211/8,658); however, the mortality in the TDM group was significantly lower than that in the non-TDM group (23.3% vs. 27.7%, *p* < 0.001). Moreover, compared with the non-TDM group, the TDM group had significantly lower ICU mortality (13.1% vs. 18.7%, *p* < 0.001) and in-hospital mortality (18.8% vs. 23.4%, *p* < 0.001). Consistent with pre-PSM results, the ICU stay and total hospital stay were significantly longer in the TDM group compared with the non-TDM group. The median ICU stay was 5.6 days (IQR 3.1–10.6) in the TDM group vs. 2.6 days (IQR 1.5–4.7) in the non-TDM group, and the median total hospital stay was 13.0 days (IQR 8.0–21.2) vs. 7.9 days (IQR 4.7–12.8), respectively (*p* < 0.001 for both comparisons).

### 3.3 Association between vancomycin TDM and 30-day mortality

**Figure 2:**
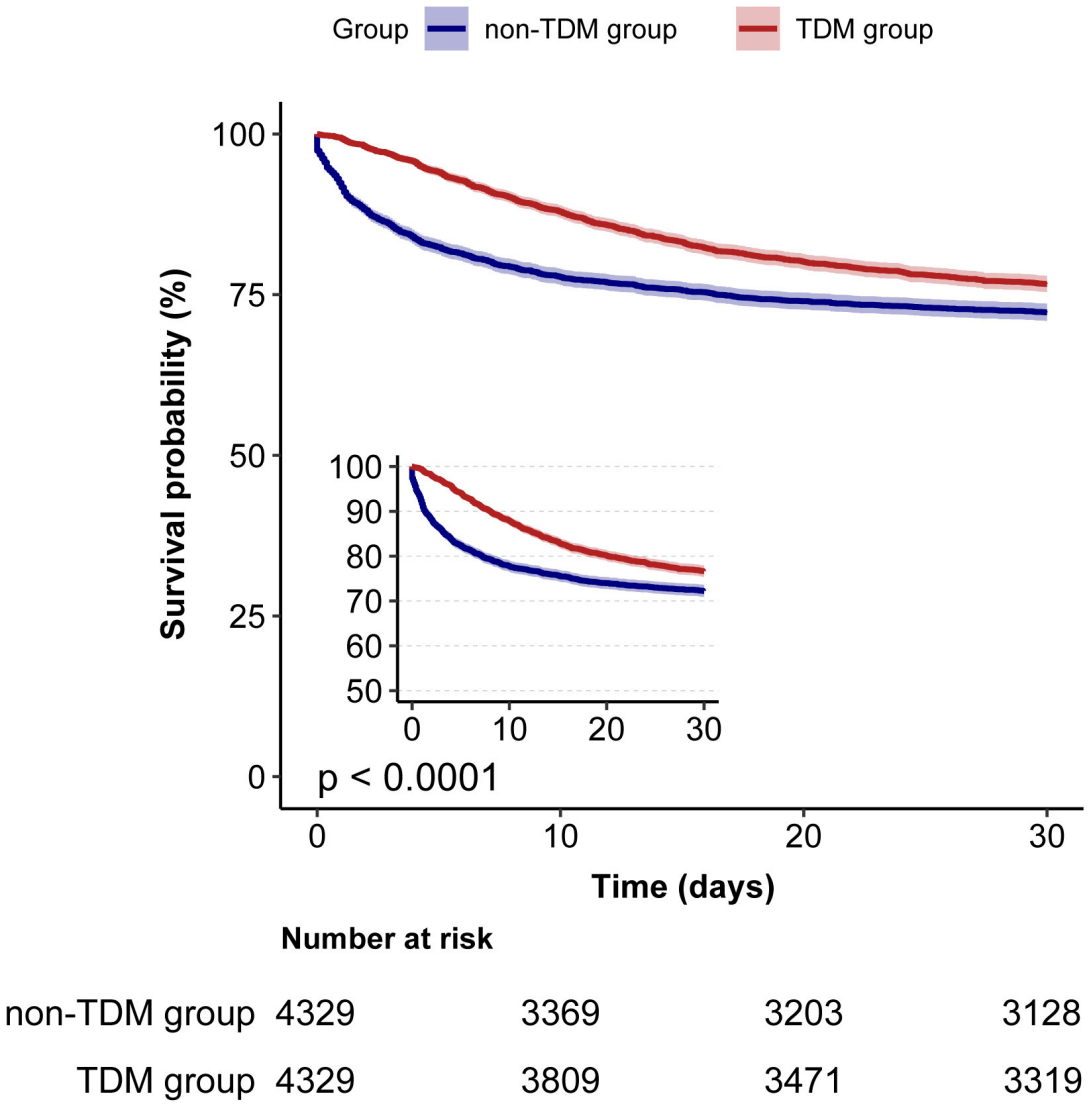
Kaplan–Meier survival curves for 30-day mortality in sepsis patients: TDM group vs. non-TDM group.

We used Cox proportional hazards regression, adjusted for all covariates in Table 1, to assess the association between vancomycin TDM and 30-day mortality. Prior to PSM, vancomycin TDM was significantly associated with lower 30-day mortality (adjusted HR: 0.66; 95% CI: 0.61–0.71; *p* < 0.001). After PSM, the association remained significant, with an adjusted HR of 0.71 (95% CI: 0.66–0.77; *p* < 0.001). Consistently, using inverse probability of treatment weighting (IPTW) based on propensity scores for further covariate adjustment, the adjusted HR was 0.72 (95% CI: 0.67–0.77; *p* < 0.001). These findings consistently demonstrate that vancomycin TDM is associated with reduced 30-day mortality, even after adjusting for potential confounders using PSM and IPTW (Table 3). In the matched cohort, the Kaplan–Meier survival curves demonstrated a significantly reduced 30-day mortality rate in the TDM group (log-rank test: *p* < 0.0001; Figure 2).

**Table 3:** The association between vancomycin TDM and 30-day mortality, as determined by analyses incorporating multiple models.

	**HR**	**95% CI**	***p*-value**
Crude analysis.Unmatched	1.29	1.21–1.39	< 0.001
Multivariable.adjusted^a^	0.66	0.61–0.71	< 0.001
PropensityScore.Matched^b^	0.76	0.70–0.83	< 0.001
PropensityScore.adjusted^c^	0.71	0.66–0.77	< 0.001
Weighted.IPTW^d^	0.72	0.67–0.77	< 0.001

^a^HR from a multivariable Cox proportional model adjusted for all covariates in Table 1.

^b^HR from a multivariate Cox proportional hazards model with the same strata and covariates matched according to the propensity score.

^c^HR from a multivariable Cox proportional hazards model with the same strata and covariates, with additional adjustment for the propensity score.

^d^HR from a multivariable Cox proportional hazards model with the same strata and covariates, with IPTW adjustment according to the propensity score.

### 3.4 Subgroup analysis

**Figure 3:**
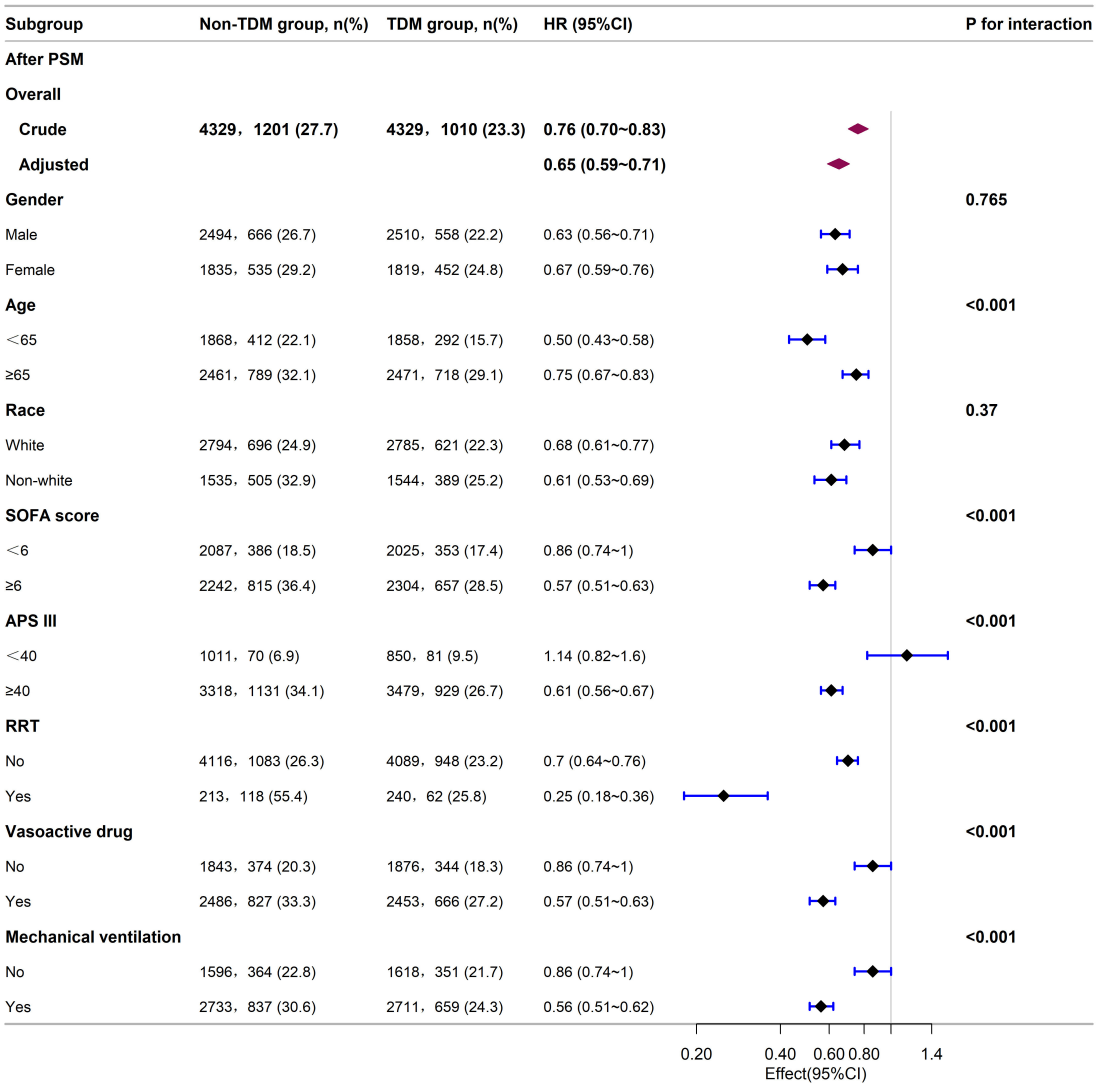
Subgroup analysis of the relationship between vancomycin TDM and 30-day mortality in sepsis patients, as visualized by a forest plot. TDM, therapeutic drug monitoring; HR, hazard ratio; CI, confidence interval; SOFA score, sequential organ failure assessment score; APS III, acute physiology score III; RRT, renal replacement therapy.

Next, we stratified the study population into clinical subgroups based on factors such as gender, age, race, SOFA score, APS III score, and the use of RRT, vasopressors, or mechanical ventilation. We evaluated the impact of vancomycin TDM on 30-day mortality in each subgroup, and presented the results in a forest plot (Figure 3). Subgroup analysis showed that vancomycin TDM was significantly associated with a lower 30-day mortality (HR < 1) across all subgroups examined, including those defined by gender, age (< 65 or ≥65 years), race (White or non-White), SOFA score (< 6 or ≥6), and the need for RRT, vasopressors, or mechanical ventilation.

Notably, the reduction in mortality risk with vancomycin TDM was more pronounced in certain subgroups: patients younger than 65 years (HR: 0.50; 95% CI: 0.43–0.58), those with a SOFA score ≥6 (HR: 0.57; 95% CI: 0.51–0.63), an acute physiology score (APS) III score ≥40 (HR: 0.61; 95% CI: 0.56–0.67), patients receiving RRT (HR: 0.25; 95% CI: 0.18–0.36), those using vasopressors (HR: 0.57; 95% CI: 0.51–0.63), and those requiring mechanical ventilation (HR: 0.56; 95% CI: 0.51–0.62). These patients experienced a greater survival benefit from vancomycin TDM compared with patients aged ≥65 years (HR: 0.75; 95% CI: 0.67–0.83), those with a SOFA score < 6 (HR: 0.86; 95% CI: 0.74–1.00), those not receiving RRT (HR: 0.70; 95% CI: 0.64–0.76), those not using vasopressors (HR: 0.86; 95% CI: 0.74–1.00), and those not requiring mechanical ventilation (HR: 0.86; 95% CI: 0.74–1.00). However, patients with an APS III score < 40 (HR: 1.14; 95% CI: 0.82–1.60) did not show a statistically significant benefit from vancomycin TDM.

### 3.5 Sensitivity analysis

To verify the robustness of our results, we conducted three sensitivity analyses (Table 4). First, excluding 3,821 patients with an ICU stay of < 48 h, analysis of the remaining 10,232 patients showed that vancomycin TDM remained associated with lower 30-day mortality (adjusted HR: 0.87; 95% CI: 0.79–0.95; *p* < 0.001). Second, after excluding 1,135 patients who tested positive for MRSA, analysis of the remaining 12,918 patients also revealed that vancomycin TDM was associated with lower 30-day mortality (adjusted HR: 0.66; 95% CI: 0.61–0.72; *p* < 0.001). This finding suggests that empirical use of vancomycin with TDM can improve outcomes in sepsis patients, even when the etiological diagnosis is unclear. Finally, after excluding patients with missing lactate values, analysis of 4,398 patients diagnosed with septic shock yielded results consistent with those observed in the overall sepsis population (adjusted HR: 0.58; 95% CI: 0.51–0.66; *p* < 0.001).

**Table 4:** Sensitivity analysis of the relationship between vancomycin TDM and 30-day mortality.

**Sensitivity**	**Matching**	**30-day mortality**, ***n*** **(%)**				**Cox proportional hazards regression analysis**		
		**Total**	**Non-TDM group**	**TDM group**	***p***	**HR**	**95%CI**	***p***
Model 1 (*n* = 10,232)	Before PSM	2,481/10,232 (24.2%)	791/3,933 (20.1%)	1,690/6,299 (26.8%)	< 0.001	0.87^a^	0.79–0.95	< 0.001
	After PSM	1,355/6,398 (21.2%)	746/3,199 (23.3%)	609/3,199 (19.0%)	< 0.001	0.69^b^	0.62–0.77	< 0.001
Model 2 (*n* = 12,918)	Before PSM	3,044/12,918 (23.6%)	1,387/6,804 (20.4%)	1,657/6,114 (27.1%)	< 0.001	0.66^a^	0.61–0.72	< 0.001
	After PSM	2,117/7,978 (26.5%)	1,105/3,989 (27.7%)	1,012/3,989 (25.4%)	< 0.001	0.62^b^	0.57–0.68	< 0.001
Model 3 (*n* = 4,398)	Before PSM	1,412/4,398 (32.1%)	593/2,110 (28.1%)	819/2,288 (35.8%)	< 0.001	0.58^a^	0.51–0.66	< 0.001
	After PSM	907/2,408 (37.7%)	493/1,204 (40.9%)	414/1,201 (34.4%)	< 0.001	0.45^b^	0.39–0.51	< 0.001

Model 1: Excluded those who ICU stay was < 48 h; Model 2: Excluded those who had positive microbiological cultures of MRSA; Model 3: Only septic shock patients were included, with the diagnostic criteria based on “The Third International Consensus Definitions for Sepsis and Septic Shock (Sepsis-3).” Patients with missing lactate data were excluded.

^a^HR from a multivariable Cox proportional model adjusted for all covariates in Table 1.

^b^HR from a multivariable Cox proportional hazards model with the same strata and covariates, with additional adjustment for the propensity score.

### 3.6 Age response relationship between vancomycin TDM and 30-day mortality

**Figure 4:**
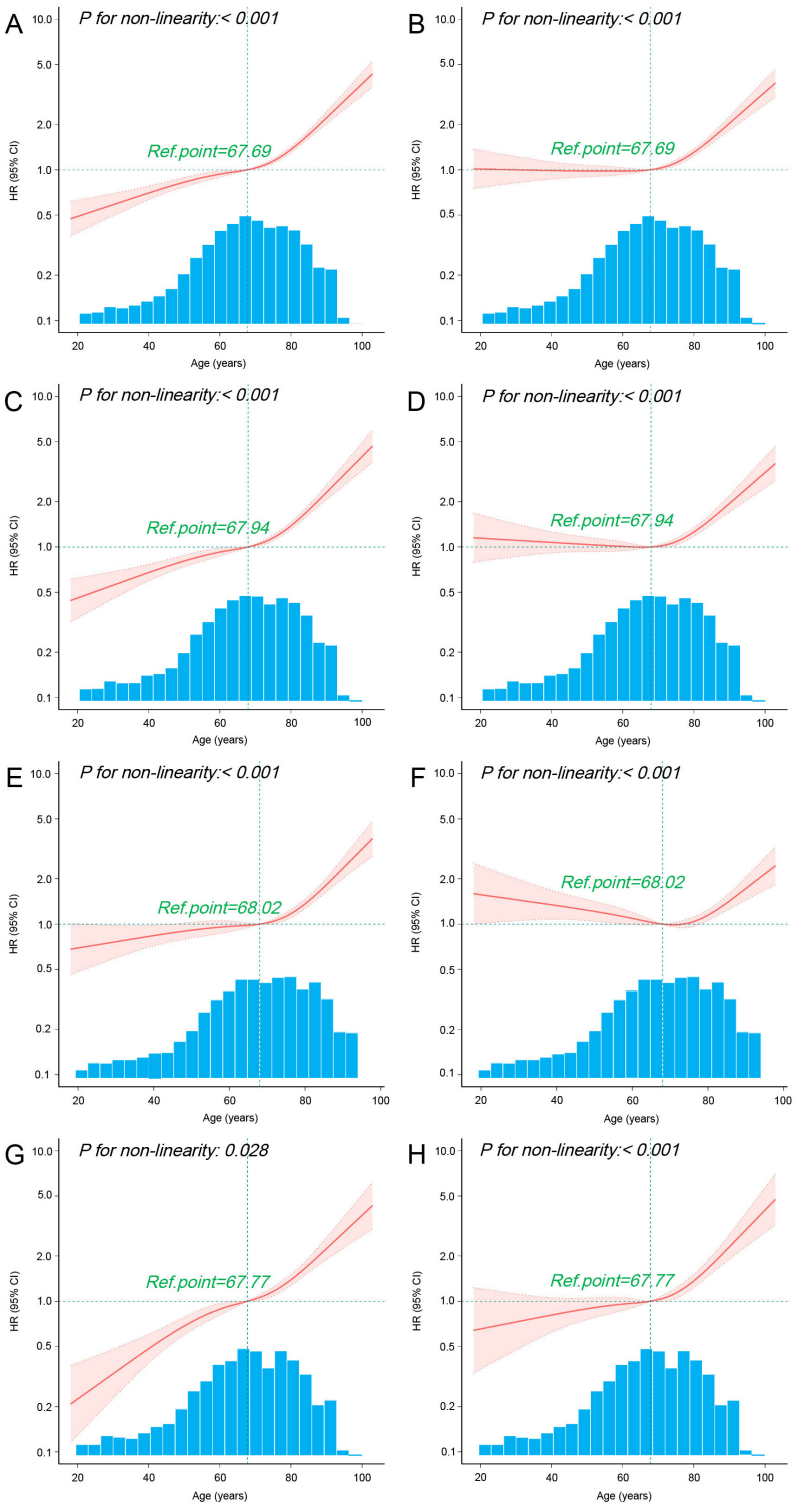
Association between age and 30-day mortality using RCS. The curves illustrate the relationship between HR and age using univariate **(A)** and multivariate **(B)** Cox regression models with RCS in all sepsis patients before PSM. Similarly, curves for HR and age are presented for univariate **(C)** and multivariate **(D)** Cox regression models with RCS in all sepsis patients after PSM. Additional analyses include univariate **(E)** and multivariate **(F)** Cox regression models with RCS in non-TDM patients after PSM, as well as univariate **(G)** and multivariate **(H)** Cox regression models with RCS in TDM patients after PSM. The multivariate Cox regression models were adjusted for all variables listed in Table 1. The cubic spline curves are shown as solid lines, with shaded areas representing the 95% confidence intervals. The “Ref.point” represents the reference age at which the HR equals 1. The hazard ratio spline is plotted on a logarithmic scale across the age distribution.

Given that age is a continuous variable, we first performed a nonlinear test to assess the relationship between age and 30-day mortality. The results indicated that both before and after PSM, the univariate and multivariate Cox regression analyses combined with RCS demonstrated a significant nonlinear relationship between age and 30-day mortality in sepsis patients (*p* < 0.05; Figures 4A–D). Further post-PSM analysis revealed a significant nonlinear relationship between age and 30-day mortality in both the TDM and non-TDM groups (*p* < 0.05; Figures 4E–H). The RCS curves from the multivariate Cox regression analyses indicated that, compared with the non-TDM group, patients in the TDM group had a lower risk of mortality (HR < 1) at ages below 67.77 years (Figures 4F, H).

**Figure 5:**
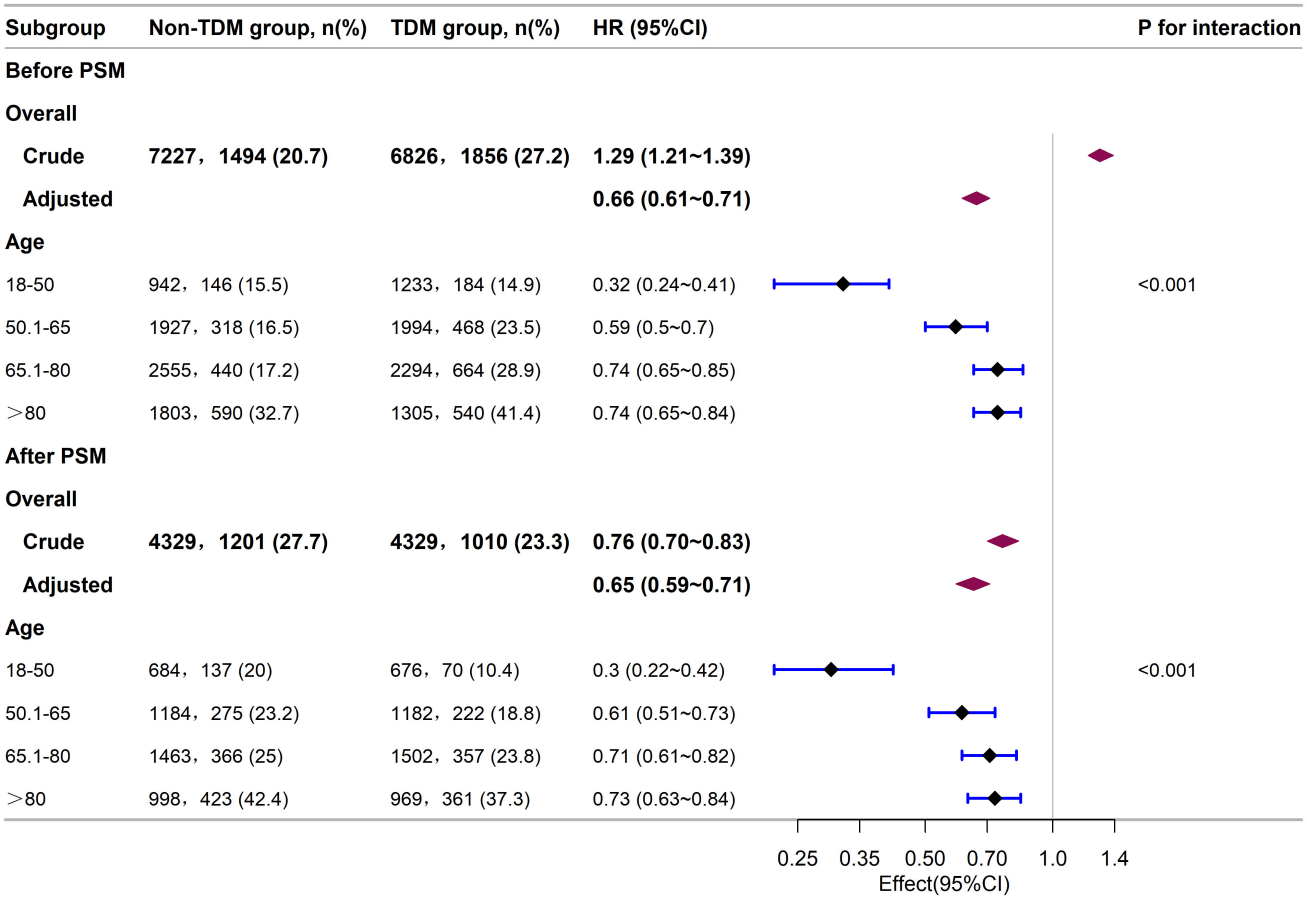
Subgroup analysis of the relationship between vancomycin TDM and 30-day mortality in sepsis patients of different age groups, as visualized by a forest plot.

**Figure 6:**
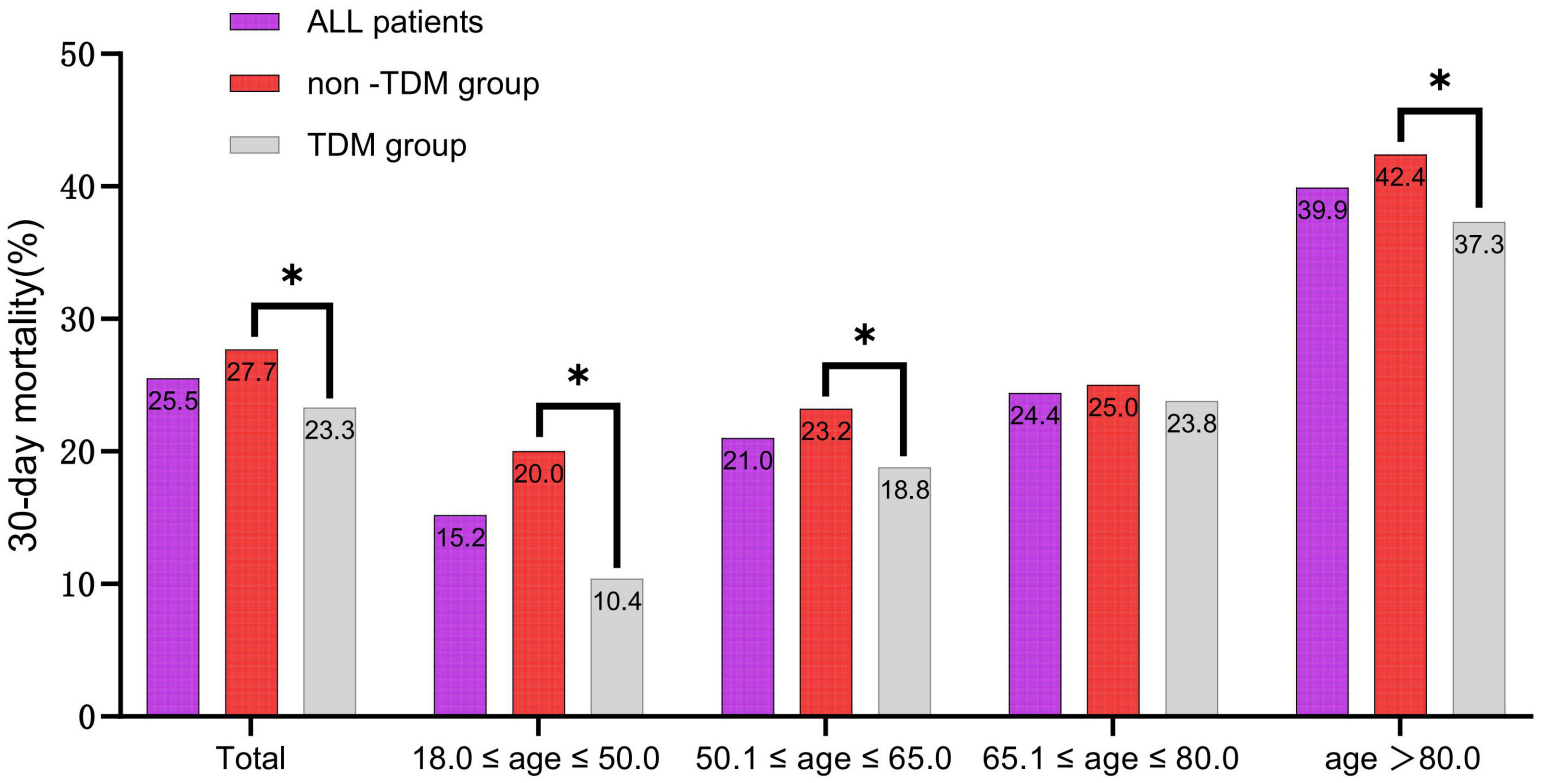
The 30-day mortality rates of sepsis patients among different age subgroups after PSM. ^*^p < 0.05.

To further analyze the impact of vancomycin TDM on 30-day mortality across different age groups, we divided the patients into four age groups: 18–50, 50.1–65, 65.1–80, and >80 years. Baseline characteristics of each group are detailed in Supplementary Tables 2, 3. Subgroup analysis suggested that both before and after PSM, sepsis patients aged 18–50 years derived the greatest survival benefit from vancomycin TDM (Figure 5). After PSM, the 18–50 age group exhibited the lowest mortality rate (15.2%), and the reduction in mortality was particularly significant among patients who received TDM compared with those who did not (Figure 6).

## 4 Discussion

In this large retrospective cohort study, we found that vancomycin TDM is significantly associated with a reduction in 30-day mortality among sepsis patients, and this effect appears to vary across different age groups. Initially, we observed a higher 30-day mortality rate in the TDM group compared to the non-TDM group. However, after adjusting the baseline differences through PSM, the trend reversed, suggesting that TDM may play a critical role in improving survival. This finding was further supported by Cox proportional hazards regression analysis, PSM-adjusted analysis, IPTW analysis, and Kaplan–Meier survival curves, all of which consistently indicated better outcomes with TDM. These results underscore the potential clinical benefits of TDM for optimizing vancomycin therapy in sepsis patients.

The association between aging and increased mortality in sepsis is well documented (25, 31). In our study, both the RCS curves before and after PSM (Figures 4A, C) and the subgroup analysis (Figure 5) also reflected this trend. Further exploration of the effect of vancomycin TDM on age-related sepsis mortality showed that while vancomycin TDM was associated with lower 30-day mortality across all age groups, the survival benefit was more pronounced in patients younger than 65 years, particularly those aged 18–50 years. This age-related heterogeneity in treatment effect may be attributed to differences in physiological resilience and comorbidity burden. Younger patients generally have stronger cardiovascular and renal function, which can enhance drug clearance and reduce the risk of toxicity. Additionally, they often exhibit stronger immune responses, which may complement the effects of optimized antibiotic therapy (32, 33). In contrast, older patients often face more complex clinical conditions, including multiple comorbidities and higher risks of resistant infections, potentially limiting the overall impact of TDM despite its ability to optimize vancomycin dosing (31). These findings highlight the importance of individualized treatment strategies that account for patient-specific factors such as age and overall health status. For example, younger patients with better renal function may benefit from more aggressive dosing strategies, while older patients with multiple comorbidities may require more conservative dosing to minimize toxicity risks. Notably, our analysis revealed that younger patients derived greater benefits from TDM, suggesting that timely and precise dosing adjustments can have a substantial impact on survival outcomes in this group. Due to their lower mortality rate, younger sepsis patients are often overlooked for TDM in clinical practice. Given the significant advantage in reducing mortality risk, our study stresses the need to implement TDM for younger sepsis patients (aged 18–50 years).

Sepsis-induced changes in volume of distribution and creatinine clearance can lead to significant fluctuations in serum drug levels, such as vancomycin, highlighting the necessity of individualized dosing strategies (34–36). The Surviving Sepsis Campaign also emphasizes the importance of optimizing antimicrobial dosing based on PK, PD, and antimicrobial resistance profiles (8). Compared to a standardized dosing approach, individualized antimicrobial dosing is better suited for sepsis patients, with TDM being a critical method for achieving optimal dosing (10, 15). Previous studies on vancomycin TDM in sepsis have primarily focused on two areas. The first is the pharmacokinetics of vancomycin in specific sepsis patient subgroups (11, 13, 37), and the second is the relationship between vancomycin trough concentrations or AUC/MIC ratios and clinical outcomes (38–41). However, only limited studies have reported on the mortality of sepsis patients related to vancomycin TDM (42, 43). The relationship between vancomycin TDM indicators and mortality in sepsis patients remains complex. According to the latest meta-analysis, while lower vancomycin trough levels are associated with a reduced risk of treatment failure and all-cause mortality, no significant correlation has been found between vancomycin trough levels and clinical outcomes in adult patients with sepsis or Gram-positive bacterial infections (44). Additionally, there remains a lack of robust data linking vancomycin AUC to mortality (45, 46). Welder-Zamone et al. identified vancomycin serum concentration as a key predictor of acute kidney injury (AKI) in sepsis patients. In their Cox regression model, a serum concentration exceeding 21.5 mg/L was the sole variable significantly associated with mortality, although the study's sample size was limited to 135 patients (47). Another study involving 3,146 elderly ICU patients found that vancomycin trough concentrations were significantly associated with AKI and increased 30-day mortality risk, with a trough concentration above 19.17 mg/L significantly elevating this risk (48). However, because the study population was limited to elderly ICU patients, the findings may not generalize to the broader sepsis population. To date, there are almost no studies that have directly compared the clinical outcomes of TDM vs. non-TDM approaches in sepsis patients. Due to the limitations of current studies, it remains unclear whether TDM significantly impacts mortality compared to non-TDM approaches in sepsis patients. Our study highlights the potential substantial benefit of vancomycin TDM in reducing mortality among sepsis patients. By leveraging a larger, more heterogeneous cohort of ICU-admitted sepsis patients, our findings may be more broadly applicable. These findings contribute to the growing body of evidence endorsing routine TDM for optimizing vancomycin therapy, particularly in critical care settings. This aligns with current guidelines that prioritize individualized dosing strategies to improve therapeutic outcomes.

This study has several limitations. As a retrospective study, it inherently carries the risk of information bias and confounding bias. Although we applied the PSM method and conducted subgroup and sensitivity analyses to reduce these biases, unmeasured confounders could still influence the results. Furthermore, retrospective studies establish associations rather than causality. Therefore, randomized controlled trials are needed to validate the survival benefits associated with vancomycin TDM in sepsis patients and to identify the subpopulations that benefit most. Future studies should adopt a multicenter design and stratify patients by age and comorbidities to clarify the differential impacts of TDM. Additionally, the data utilized in this study were drawn from the single-center MIMIC-IV database, potentially limiting the generalizability of our findings. Moreover, implementing TDM necessitates specialized equipment, trained personnel, and entails additional time and costs. While our study suggested that vancomycin TDM reduced mortality risk in sepsis patients, the cost-effectiveness of routine TDM implementation across all patients remains uncertain.

## 5 Conclusions

This retrospective analysis revealed that vancomycin TDM was associated with a reduction in 30-day mortality among sepsis patients, with the most significant impact observed in those aged 18–50 years. Despite the limitations inherent in the retrospective design and potential confounding factors, the findings support the implementation of TDM as a standard practice for vancomycin therapy in sepsis patients across all age groups. Furthermore, the study found that although younger sepsis patients exhibited lower mortality rates, vancomycin TDM conferred a greater benefit in enhancing their survival.

## Data Availability

The original contributions presented in the study are included in the article/Supplementary material, further inquiries can be directed to the corresponding authors.

## Supplementary material

Supplementary Table 4 The baseline characteristics of the patients enrolled from the MIMIC-IV database (including p-values).
