# Supplementary material for: Impact of vancomycin therapeutic drug monitoring on mortality in sepsis patients across different age groups: a propensity score-matched retrospective cohort study
Source: Front Med (Lausanne). 2024 Dec 12;11:1498337. doi: 10.3389/fmed.2024.1498337 (PMC11669523; doi:10.3389/fmed.2024.1498337)
Supplement: Supplementary file 1 [file Table_1.DOCX]

Supplementary Table S1：Detailed information on missing data of variables in the study

| variable | inventory | N missing | missing rate |
| --- | --- | --- | --- |
| Heart rate | 14030 | 23 | 0.16% |
| MAP | 14030 | 23 | 0.16% |
| Respiratory rate | 14026 | 27 | 0.19% |
| Temperature | 13381 | 672 | 4.78% |
| Spo2 | 14026 | 27 | 0.19% |
| WBC | 14025 | 28 | 0.20% |
| Hemoglobin | 14024 | 29 | 0.21% |
| Hematocrit | 14030 | 23 | 0.16% |
| Platelets | 14026 | 27 | 0.19% |
| Creatinine | 14037 | 16 | 0.11% |
| BUN | 14030 | 23 | 0.16% |
| Glucose | 14002 | 51 | 0.36% |
| Potassium | 14021 | 32 | 0.23% |
| Bicarbonate | 14031 | 22 | 0.16% |
| SOFA score | 14031 | 22 | 0.16% |
| Lactate | 9840 | 4213 | 29.98% |

The other variables in this study do not missing.
