# Supplementary material for: Impact of vancomycin therapeutic drug monitoring on mortality in sepsis patients across different age groups: a propensity score-matched retrospective cohort study
Source: Front Med (Lausanne). 2024 Dec 12;11:1498337. doi: 10.3389/fmed.2024.1498337 (PMC11669523; doi:10.3389/fmed.2024.1498337)
Supplement: Supplementary file 2 [file Table_2.docx]

Supplementary Table 2：Baseline characteristics of sepsis patients across different age groups after PSM

| **Patient**  **characteristic** | **After PSM** | | | | | |
| --- | --- | --- | --- | --- | --- | --- |
|  | Total (n = 8658) | 18-50 years  (n = 1360) | 50.1-65 years (n = 2366) | 65.1-80 years (n = 2965) | >80 years (n = 1967) | *p* |
| Gender[male, n(%)] | 5004 (57.8) | 814 (59.9) | 1490 (63) | 1708 (57.6) | 992 (50.4) | < 0.001 |
| Age(years) | 66.2 ± 16.5 | 37.9 ± 8.8 | 58.3 ± 4.2 | 72.4 ± 4.3 | 86.1 ± 4.0 | < 0.001 |
| RACE[white, n(%)] | 5579 (64.4) | 698 (51.3) | 1535 (64.9) | 1946 (65.6) | 1400 (71.2) | < 0.001 |
| **Vital signs** | | | | | | |
| Heart rate(bpm) | 88.4 ± 16.6 | 94.2 ± 17.2 | 89.8 ± 16.5 | 86.5 ± 15.9 | 85.4 ± 16.0 | < 0.001 |
| MAP(mmHg) | 76.3 ± 10.4 | 79.7 ± 11.2 | 77.9 ± 10.7 | 75.5 ± 9.7 | 73.2 ± 9.5 | < 0.001 |
| Respiratory rate(/min) | 20.2 ± 4.2 | 20.8 ± 4.7 | 20.0 ± 4.3 | 19.9 ± 3.9 | 20.6 ± 4.0 | < 0.001 |
| Temperature(°C) | 37.6 ± 0.9 | 37.9 ± 1.0 | 37.6 ± 0.9 | 37.5 ± 0.8 | 37.4 ± 0.8 | < 0.001 |
| SpO2(%) | 96.8 ± 2.6 | 97.1 ± 2.7 | 96.7 ± 2.8 | 96.8 ± 2.5 | 96.7 ± 2.3 | < 0.001 |
| **Laboratory tests** | | | | | | |
| WBC(×109 ) | 14.6 (10.3, 19.9) | 15.0 (10.4, 20.8) | 14.2 (9.9, 19.6) | 14.6 (10.5, 19.5) | 14.7 (10.6, 20.3) | 0.003 |
| Hemoglobin(g/L) | 9.9 ± 2.2 | 10.2 ± 2.4 | 10.0 ± 2.3 | 9.7 ± 2.1 | 9.7 ± 2.0 | < 0.001 |
| Hematocrit(%) | 29.9 ± 6.6 | 30.5 ± 7.0 | 30.0 ± 6.8 | 29.5 ± 6.4 | 29.9 ± 6.1 | < 0.001 |
| Platelets(×109 ) | 164.0 (109.0, 232.0) | 168.0 (109.8, 232.0) | 160.0 (102.0, 230.0) | 162.0 (111.0, 229.0) | 170.0 (115.5, 235.0) | < 0.001 |
| Creatinine(mg/dL) | 1.2 (0.9, 2.0) | 1.0 (0.8, 1.5) | 1.2 (0.8, 1.9) | 1.3 (0.9, 2.1) | 1.4 (1.0, 2.1) | < 0.001 |
| BUN(mg/dL) | 24.0 (16.0, 41.0) | 16.0 (11.0, 25.0) | 22.0 (15.0, 36.0) | 27.0 (18.0, 43.0) | 32.0 (22.0, 49.0) | < 0.001 |
| Glucose (finger,mg/dL) | 134.7 (114.7, 167.3) | 124.5 (106.0, 150.6) | 135.8 (116.0, 171.5) | 138.1 (118.5, 172.3) | 135.0 (114.4, 166.3) | < 0.001 |
| Potassium(mmol/L) | 3.9 ± 0.6 | 3.7 ± 0.6 | 3.9 ± 0.6 | 3.9 ± 0.6 | 3.9 ± 0.6 | < 0.001 |
| Bicarbonate(mmol/L) | 20.6 ± 5.2 | 20.3 ± 5.1 | 20.5 ± 5.3 | 20.7 ± 5.2 | 20.6 ± 5.3 | 0.074 |
| **Comorbidity diseases, n(%)** | | | | | | |
| Hypertension | 5388 (62.2) | 370 (27.2) | 1346 (56.9) | 2164 (73) | 1508 (76.7) | < 0.001 |
| Congestive heart failure | 2707 (31.3) | 162 (11.9) | 522 (22.1) | 1072 (36.2) | 951 (48.3) | < 0.001 |
| COPD | 2334 (27.0) | 211 (15.5) | 565 (23.9) | 954 (32.2) | 604 (30.7) | < 0.001 |
| Liver disease | 1400 (16.2) | 294 (21.6) | 601 (25.4) | 380 (12.8) | 125 (6.4) | < 0.001 |
| Diabetes | 2117 (24.5) | 155 (11.4) | 589 (24.9) | 889 (30) | 484 (24.6) | < 0.001 |
| Renal disease | 2004 (23.1) | 119 (8.8) | 390 (16.5) | 793 (26.7) | 702 (35.7) | < 0.001 |
| Malignant cancer | 1298 (15.0) | 93 (6.8) | 389 (16.4) | 541 (18.2) | 275 (14) | < 0.001 |
| Cerebrovascular disease | 1325 (15.3) | 163 (12) | 331 (14) | 486 (16.4) | 345 (17.5) | < 0.001 |
| **Severity of illness scores** | | | | | | |
| CCI | 6.0 ± 3.0 | 2.3 ± 2.2 | 5.5 ± 2.5 | 7.2 ± 2.5 | 7.4 ± 2.3 | < 0.001 |
| SOFA score | 6.0 (4.0, 8.0) | 5.0 (3.0, 7.2) | 6.0 (4.0, 8.0) | 6.0 (4.0, 8.0) | 6.0 (4.0, 8.0) | < 0.001 |
| APS III | 60.0 ± 24.8 | 55.3 ± 25.6 | 58.1 ± 25.5 | 60.3 ± 24.3 | 65.1 ± 23.1 | < 0.001 |
| SAPS II | 41.9 ± 15.1 | 30.9 ± 13.9 | 38.6 ± 14.3 | 44.5 ± 13.5 | 49.7 ± 13.4 | < 0.001 |
| OASIS | 36.6 ± 8.9 | 33.4 ± 8.6 | 35.2 ± 8.7 | 36.9 ± 8.6 | 40.0 ± 8.4 | < 0.001 |
| **Therapy, n(%)** | | | | | | |
| RRT | 453 ( 5.2) | 83 (6.1) | 125 (5.3) | 165 (5.6) | 80 (4.1) | 0.043 |
| Mechanical ventilation | 5444 (62.9) | 913 (67.1) | 1527 (64.5) | 1892 (63.8) | 1112 (56.5) | < 0.001 |
| Vasoactive drug | 4939 (57.0) | 603 (44.3) | 1369 (57.9) | 1810 (61) | 1157 (58.8) | < 0.001 |
| **Infectious pathogen, n (%)** | | | | | | |
| MRSA | 708 ( 8.2) | 100 (7.4) | 163 (6.9) | 239 (8.1) | 206 (10.5) | < 0.001 |
