# Supplementary material for: Impact of vancomycin therapeutic drug monitoring on mortality in sepsis patients across different age groups: a propensity score-matched retrospective cohort study
Source: Front Med (Lausanne). 2024 Dec 12;11:1498337. doi: 10.3389/fmed.2024.1498337 (PMC11669523; doi:10.3389/fmed.2024.1498337)
Supplement: Supplementary file 3 [file Table_3.docx]

Supplementary Table 3：Baseline characteristics of sepsis patients across different age groups before PSM

| **Patient**  **characteristic** | **Before PSM** | | | | | |
| --- | --- | --- | --- | --- | --- | --- |
|  | Total (n = 14053) | 18-50 years  (n = 2175) | 50.1-65 years (n = 3921) | 65.1-80 years (n = 4849) | >80 years (n = 3108) | *p* |
| Gender[male, n(%)] | 8197 (58.3) | 1315 (60.5) | 2489 (63.5) | 2846 (58.7) | 1547 (49.8) | < 0.001 |
| Age(years) | 66.2 ± 16.3 | 38.4 ± 8.7 | 58.3 ± 4.2 | 72.3 ± 4.3 | 86.2 ± 4.1 | < 0.001 |
| RACE[white, n(%)] | 9168 (65.2) | 1118 (51.4) | 2551 (65.1) | 3260 (67.2) | 2239 (72) | < 0.001 |
| **Vital signs** | | | | | | |
| Heart rate(bpm) | 88.2 ± 16.5 | 94.8 ± 17.6 | 89.4 ± 16.2 | 86.3 ± 15.5 | 85.0 ± 16.0 | < 0.001 |
| MAP(mmHg) | 76.1 ± 10.2 | 79.5 ± 11.3 | 77.6 ± 10.3 | 75.3 ± 9.3 | 73.1 ± 9.6 | < 0.001 |
| Respiratory rate(/min) | 20.2 ± 4.2 | 21.0 ± 4.8 | 20.0 ± 4.3 | 19.8 ± 3.9 | 20.5 ± 4.0 | < 0.001 |
| Temperature(°C) | 37.6 ± 0.9 | 37.9 ± 1.0 | 37.6 ± 0.9 | 37.5 ± 0.8 | 37.4 ± 0.8 | < 0.001 |
| SpO2(%) | 96.8 ± 2.6 | 97.1 ± 2.5 | 96.7 ± 2.8 | 96.9 ± 2.4 | 96.7 ± 2.3 | < 0.001 |
| **Laboratory tests** | | | | | | |
| WBC(×10^9^ ) | 14.5 (10.3, 19.8) | 15.1 (10.4, 20.8) | 14.3 (10.0, 19.5) | 14.6 (10.5, 19.6) | 14.2 (10.4, 19.7) | < 0.001 |
| Hemoglobin(g/L) | 9.9 ± 2.2 | 10.3 ± 2.4 | 9.9 ± 2.2 | 9.7 ± 2.1 | 9.7 ± 2.0 | < 0.001 |
| Hematocrit(%) | 29.8 ± 6.5 | 30.6 ± 7.0 | 29.8 ± 6.6 | 29.4 ± 6.3 | 29.8 ± 6.1 | < 0.001 |
| Platelets(×10^9^ ) | 161.0 (108.0, 227.0) | 165.0 (107.0, 230.5) | 154.0 (101.0, 222.0) | 160.0 (111.0, 224.0) | 168.0 (115.0, 233.0) | < 0.001 |
| Creatinine(mg/dL) | 1.2 (0.9, 2.0) | 1.1 (0.8, 1.8) | 1.1 (0.8, 1.9) | 1.2 (0.9, 2.0) | 1.3 (1.0, 2.0) | < 0.001 |
| BUN(mg/dL) | 24.0 (16.0, 40.0) | 17.0 (12.0, 28.0) | 21.0 (15.0, 37.0) | 25.0 (17.0, 42.0) | 30.5 (21.0, 47.0) | < 0.001 |
| Glucose (finger,mg/dL) | 133.4 (114.8, 164.3) | 125.6 (106.8, 153.0) | 134.3 (115.8, 169.1) | 136.2 (118.7, 168.8) | 132.6 (113.3, 161.2) | < 0.001 |
| Potassium(mmol/L) | 3.9 ± 0.6 | 3.8 ± 0.6 | 3.9 ± 0.6 | 3.9 ± 0.6 | 3.9 ± 0.6 | < 0.001 |
| Bicarbonate(mmol/L) | 20.6 ± 5.1 | 20.1 ± 5.2 | 20.6 ± 5.2 | 20.8 ± 5.0 | 20.7 ± 5.2 | < 0.001 |
| **Comorbidity diseases, n(%)** | | | | | | |
| Hypertension | 8776 (62.4) | 655 (30.1) | 2194 (56) | 3549 (73.2) | 2378 (76.5) | < 0.001 |
| Congestive heart failure | 4249 (30.2) | 274 (12.6) | 827 (21.1) | 1694 (34.9) | 1454 (46.8) | < 0.001 |
| COPD | 3729 (26.5) | 353 (16.2) | 943 (24) | 1518 (31.3) | 915 (29.4) | < 0.001 |
| Liver disease | 2307 (16.4) | 507 (23.3) | 989 (25.2) | 609 (12.6) | 202 (6.5) | < 0.001 |
| Diabetes | 3463 (24.6) | 280 (12.9) | 980 (25) | 1451 (29.9) | 752 (24.2) | < 0.001 |
| Renal disease | 3103 (22.1) | 201 (9.2) | 608 (15.5) | 1239 (25.6) | 1055 (33.9) | < 0.001 |
| Malignant cancer | 2048 (14.6) | 147 (6.8) | 627 (16) | 831 (17.1) | 443 (14.3) | < 0.001 |
| Cerebrovascular disease | 2039 (14.5) | 254 (11.7) | 522 (13.3) | 748 (15.4) | 515 (16.6) | < 0.001 |
| **Severity of illness scores** | | | | | | |
| CCI | 5.9 ± 2.9 | 2.5 ± 2.3 | 5.4 ± 2.5 | 7.0 ± 2.5 | 7.3 ± 2.3 | < 0.001 |
| SOFA score | 6.0 (4.0, 8.0) | 5.0 (3.0, 9.0) | 6.0 (4.0, 9.0) | 6.0 (4.0, 8.0) | 6.0 (4.0, 8.0) | < 0.001 |
| APS III | 60.4 ± 27.2 | 60.2 ± 29.3 | 59.5 ± 28.7 | 59.6 ± 26.4 | 62.9 ± 24.5 | < 0.001 |
| SAPS II | 41.6 ± 15.1 | 32.2 ± 14.5 | 38.7 ± 14.7 | 43.7 ± 13.8 | 48.7 ± 13.4 | < 0.001 |
| OASIS | 36.4 ± 9.5 | 34.4 ± 9.5 | 35.3 ± 9.6 | 36.4 ± 9.4 | 39.0 ± 8.9 | < 0.001 |
| **Therapy, n(%)** | | | | | | |
| RRT | 856 ( 6.1) | 182 (8.4) | 260 (6.6) | 292 (6) | 122 (3.9) | < 0.001 |
| Mechanical ventilation | 8620 (61.3) | 1500 (69) | 2527 (64.4) | 2996 (61.8) | 1597 (51.4) | < 0.001 |
| Vasoactive drug | 8236 (58.6) | 1093 (50.3) | 2357 (60.1) | 3033 (62.5) | 1753 (56.4) | < 0.001 |
| **Infectious pathogen, n (%)** | | | | | | |
| MRSA | 1135 ( 8.1) | 166 (7.6) | 266 (6.8) | 397 (8.2) | 306 (9.8) | < 0.001 |
