# Supplementary material for: Impact of vancomycin therapeutic drug monitoring on mortality in sepsis patients across different age groups: a propensity score-matched retrospective cohort study
Source: Front Med (Lausanne). 2024 Dec 12;11:1498337. doi: 10.3389/fmed.2024.1498337 (PMC11669523; doi:10.3389/fmed.2024.1498337)
Supplement: Supplementary Table 4 — The baseline characteristics of the patients enrolled from the MIMIC-IV database (including p-values). [file Table_4.docx]

**Supplementary Table S4.** The baseline characteristics of the patients enrolled from the MIMIC-IV database (including p-values).

| Patient  characteristic | Before PSM | | | | After PSM | | | |
| --- | --- | --- | --- | --- | --- | --- | --- | --- |
|  | Total  (n = 14053) | Non-TDM group  (n = 7227) | TDM group  (n = 6826) | *p* | Total  (n = 8658) | Non-TDM  group(n = 4329) | TDM group  (n = 4329) | *p* |
| Gender[male, n(%)] | 8197 (58.3) | 4153 (57.5) | 4044 (59.2) | 0.033 | 3654 (42.2) | 1835 (42.4) | 1819 (42) | 0.728 |
| Age(years) | 66.2 ± 16.3 | 67.8 ± 15.9 | 64.6 ± 16.5 | < 0.001 | 66.2 ± 16.5 | 66.3 ± 16.6 | 66.2 ± 16.4 | 0.799 |
| RACE[white, n(%)] | 9168 (65.2) | 4907 (67.9) | 4261 (62.4) | < 0.001 | 5579 (64.4) | 2794 (64.5) | 2785 (64.3) | 0.84 |
| Vital signs | | | | | | | | |
| Heart rate(bpm) | 88.2 ± 16.5 | 86.6 ± 15.6 | 89.8 ± 17.2 | < 0.001 | 88.4 ± 16.6 | 88.4 ± 16.4 | 88.3 ± 16.7 | 0.866 |
| MAP(mmHg) | 76.1 ± 10.2 | 75.7 ± 10.0 | 76.5 ± 10.4 | < 0.001 | 76.3 ± 10.4 | 76.3 ± 10.6 | 76.3 ± 10.3 | 0.904 |
| Respiratory rate(/min) | 20.2 ± 4.2 | 19.7 ± 4.0 | 20.8 ± 4.3 | < 0.001 | 20.2 ± 4.2 | 20.2 ± 4.2 | 20.2 ± 4.1 | 0.483 |
| Temperature(°C) | 37.6 ± 0.9 | 37.5 ± 0.8 | 37.7 ± 0.9 | < 0.001 | 37.6 ± 0.9 | 37.6 ± 0.9 | 37.6 ± 0.9 | 0.529 |
| SpO2(%) | 96.8 ± 2.6 | 96.8 ± 2.6 | 96.8 ± 2.5 | 0.903 | 96.8 ± 2.6 | 96.8 ± 2.7 | 96.8 ± 2.4 | 0.864 |
| Laboratory tests | | | | | | | | |
| WBC(×10^9^ ) | 14.5 (10.3, 19.8) | 14.2 (10.1, 19.0) | 14.9 (10.5, 20.5) | < 0.001 | 14.6 (10.3, 19.9) | 14.6 (10.4, 19.7) | 14.6 (10.3, 20.1) | 0.655 |
| Hemoglobin(g/L) | 9.9 ± 2.2 | 9.8 ± 2.1 | 9.9 ± 2.2 | 0.449 | 9.9 ± 2.2 | 9.9 ± 2.2 | 9.9 ± 2.2 | 0.876 |
| Hematocrit(%) | 29.8 ± 6.5 | 29.6 ± 6.3 | 30.0 ± 6.7 | 0.002 | 29.9 ± 6.6 | 29.9 ± 6.6 | 29.9 ± 6.6 | 0.918 |
| Platelets(×10^9^ ) | 161.0 (108.0, 227.0) | 158.0 (110.0, 220.0) | 165.0 (105.2, 233.0) | 0.007 | 164.0 (109.0, 232.0) | 164.0 (112.0, 230.0) | 164.0 (107.0, 233.0) | 0.959 |
| Creatinine(mg/dL) | 1.2 (0.9, 2.0) | 1.1 (0.8, 1.6) | 1.3 (0.9, 2.4) | < 0.001 | 1.2 (0.9, 2.0) | 1.2 (0.9, 1.9) | 1.2 (0.8, 2.0) | 0.595 |
| BUN(mg/dL) | 24.0 (16.0, 40.0) | 22.0 (15.0, 34.0) | 27.0 (17.0, 46.0) | < 0.001 | 24.0 (16.0, 41.0) | 24.0 (16.0, 40.0) | 25.0 (16.0, 41.0) | 0.964 |
| Glucose (finger,mg/dL) | 133.5 (114.8, 164.3) | 131.5 (115.0, 157.1) | 136.5 (114.5, 171.8) | < 0.001 | 134.7 (114.7, 167.3) | 134.9 (116.0, 167.4) | 134.4 (114.0, 167.2) | 0.235 |
| Potassium(mmol/L) | 3.9 ± 0.6 | 3.9 ± 0.6 | 3.9 ± 0.6 | 0.003 | 3.9 ± 0.6 | 3.9 ± 0.6 | 3.9 ± 0.6 | 0.832 |
| Bicarbonate(mmol/L) | 20.6 ± 5.1 | 21.0 ± 4.9 | 20.1 ± 5.4 | < 0.001 | 20.6 ± 5.2 | 20.5 ± 5.4 | 20.6 ± 5.1 | 0.779 |
| Comorbidity diseases, n(%) | | | | | | | | |
| Hypertension | 8776 (62.4) | 4618 (63.9) | 4158 (60.9) | < 0.001 | 5388 (62.2) | 2690 (62.1) | 2698 (62.3) | 0.859 |
| Congestive heart failure | 4249 (30.2) | 2109 (29.2) | 2140 (31.4) | 0.005 | 2707 (31.3) | 1365 (31.5) | 1342 (31) | 0.594 |
| COPD | 3729 (26.5) | 1847 (25.6) | 1882 (27.6) | 0.007 | 2334 (27.0) | 1188 (27.4) | 1146 (26.5) | 0.309 |
| Liver disease | 2307 (16.4) | 945 (13.1) | 1362 (20) | < 0.001 | 1400 (16.2) | 704 (16.3) | 696 (16.1) | 0.815 |
| Diabetes | 3463 (24.6) | 1794 (24.8) | 1669 (24.5) | 0.608 | 2117 (24.5) | 1058 (24.4) | 1059 (24.5) | 0.980 |
| Renal disease | 3103 (22.1) | 1424 (19.7) | 1679 (24.6) | < 0.001 | 2004 (23.1) | 992 (22.9) | 1012 (23.4) | 0.610 |
| Malignant cancer | 2048 (14.6) | 1063 (14.7) | 985 (14.4) | 0.64 | 1298 (15.0) | 649 (15) | 649 (15) | 1.000 |
| Cerebrovascular disease | 2039 (14.5) | 898 (12.4) | 1141 (16.7) | < 0.001 | 1325 (15.3) | 664 (15.3) | 661 (15.3) | 0.929 |
| Severity of illness scores | | | | | | | | |
| CCI | 5.9 ± 2.9 | 5.9 ± 2.9 | 6.0 ± 3.0 | 0.036 | 6.0 ± 3.0 | 6.0 ± 3.0 | 6.0 ± 3.0 | 0.714 |
| SOFA score | 6.0 (4.0, 8.0) | 5.0 (3.0, 7.0) | 7.0 (4.0, 9.0) | < 0.001 | 6.0 (4.0, 8.0) | 6.0 (4.0, 8.0) | 6.0 (4.0, 8.0) | 0.874 |
| APS III | 60.4 ± 27.2 | 52.4 ± 24.5 | 68.8 ± 27.3 | < 0.001 | 60.0 ± 24.8 | 60.0 ± 26.0 | 60.0 ± 23.5 | 0.98 |
| SAPS II | 41.6 ± 15.1 | 39.7 ± 14.7 | 43.7 ± 15.1 | < 0.001 | 41.9 ± 15.1 | 42.1 ± 15.9 | 41.8 ± 14.2 | 0.336 |
| OASIS | 36.4 ± 9.5 | 33.9 ± 9.0 | 39.0 ± 9.3 | < 0.001 | 36.6 ± 8.9 | 36.7 ± 9.0 | 36.5 ± 8.8 | 0.489 |
| Therapy, n(%) | | | | | | | | |
| RRT | 856 ( 6.1) | 231 (3.2) | 625 (9.2) | < 0.001 | 453 ( 5.2) | 213 (4.9) | 240 (5.5) | 0.193 |
| Mechanical ventilation | 8620 (61.3) | 3595 (49.7) | 5025 (73.6) | < 0.001 | 5444 (62.9) | 2733 (63.1) | 2711 (62.6) | 0.625 |
| Vasoactive drug | 8236 (58.6) | 3786 (52.4) | 4450 (65.2) | < 0.001 | 4939 (57.0) | 2486 (57.4) | 2453 (56.7) | 0.474 |
| Infectious pathogen, n (%) | | | | | | | | |
| MRSA | 1135 ( 8.1) | 423 (5.9) | 712 (10.4) | < 0.001 | 708 ( 8.2) | 351 (8.1) | 357 (8.2) | 0.814 |
